# A Scene Recognition and Semantic Analysis Approach to Unhealthy Sitting Posture Detection during Screen-Reading

**DOI:** 10.3390/s18093119

**Published:** 2018-09-16

**Authors:** Weidong Min, Hao Cui, Qing Han, Fangyuan Zou

**Affiliations:** 1 School of Information Engineering, Nanchang University, Nanchang 330031, China; hqucuihao@163.com (H.C.); hanqing@ncu.edu.cn (Q.H.); ncuzoufangyuan@163.com (F.Z.); 2 School of Software Engineering, Nanchang University, Nanchang 330029, China

**Keywords:** unhealthy sitting posture detection, deep learning, scene recognition, semantic analysis, behavioral clustering, robotic systems

## Abstract

Behavior analysis through posture recognition is an essential research in robotic systems. Sitting with unhealthy sitting posture for a long time seriously harms human health and may even lead to lumbar disease, cervical disease and myopia. Automatic vision-based detection of unhealthy sitting posture, as an example of posture detection in robotic systems, has become a hot research topic. However, the existing methods only focus on extracting features of human themselves and lack understanding relevancies among objects in the scene, and henceforth fail to recognize some types of unhealthy sitting postures in complicated environments. To alleviate these problems, a scene recognition and semantic analysis approach to unhealthy sitting posture detection in screen-reading is proposed in this paper. The key skeletal points of human body are detected and tracked with a Microsoft Kinect sensor. Meanwhile, a deep learning method, Faster R-CNN, is used in the scene recognition of our method to accurately detect objects and extract relevant features. Then our method performs semantic analysis through Gaussian-Mixture behavioral clustering for scene understanding. The relevant features in the scene and the skeletal features extracted from human are fused into the semantic features to discriminate various types of sitting postures. Experimental results demonstrated that our method accurately and effectively detected various types of unhealthy sitting postures in screen-reading and avoided error detection in complicated environments. Compared with the existing methods, our proposed method detected more types of unhealthy sitting postures including those that the existing methods could not detect. Our method can be potentially applied and integrated as a medical assistance in robotic systems of health care and treatment.

## 1. Introduction

Behavior analysis through posture recognition is an essential research topic in robotic systems. More and more researchers are keen to study behavior recognition and semantic analysis [1,2,3,4,5]. Sitting with an unhealthy sitting posture for a long time may seriously harm human health and even lead to lumbar disease, cervical disease and myopia. Automatic vision-based detection of unhealthy sitting posture, as an example of posture detection in robotic systems, has become a hot research topic. In all industrialized countries, three quarters of the population in work need to sit for a long time [6]. Although computers make our life more convenient and comfortable, they also bring some problems. Unhealthy sitting posture not only increases the risk of occupational musculoskeletal disease, i.e., lumbar disease and cervical disease but it is closely related to the incidence of myopia. According to the study by the National Institute for Occupational Safety and Health (NIOSH) on musculoskeletal disease and occupational factors, unhealthy sitting postures caused by incorrect postures of the trunk and neck are closely related to human skeletal diseases [6,7]. Lis et al. [8] found that working in an unhealthy sitting posture for more than five hours would increase the probability of contracting backache and sciatica. However, [8] also stated that the risk of contracting backache and sciatica would be less if workers could properly control or reduce the time of non-vertical sitting posture. Clinical reports [9] have indicated that a non-vertical sitting posture or other unhealthy sitting postures would cause lumbar, cervical and abdominal muscle dysfunctions, and these symptoms might further lead to the deterioration of musculoskeletal disease like for people suffering from musculoskeletal disease. For workers using Video Display Terminals (VDTs) in the workplace, the position of the VDT also affects their sitting posture. Straker et al. [10] investigated VDT workers and then found that the correlation between using VDT and musculoskeletal disease in the back or neck reached 20%. In the workplace, sitting still, even in a correct posture, for a long time without changing posture also will cause a partial muscle burden on the neck and lumbar region. Grandjean et al. [11] introduced the time of work in stationary posture in unhealthy sitting detection because prolonged and frequent work in a stationary posture led to chronic pain. Therefore, judging unhealthy sitting posture has far-reaching implications for medical assistance in health care or treatment, and can also be applied to intelligent robotic systems to improve the life quality of humans. In order to automatically detect sitting posture and remind people to maintain a healthy sitting posture, it is extremely important to achieve high-precision sitting posture detection.

Over the past decade, lots of researchers have been dedicated to the study of sitting posture detection. The sitting posture detection methods can be roughly classified into two categories, i.e., auxiliary-equipment-based methods and vision-based methods.

As for the auxiliary-equipment-based methods, Meyer et al. [12] built a model which enhanced the accuracy of textile sensors of commercial, non-textile pressure sensing mats, then applied the sensor system to classification of sitting posture in a chair. Mattmann et al. [13] used a thermoplastic elastomer strain sensor to measure strain in clothes, and then identified different postures by Naïve Bayes classification. Ma et al. [14] proposed a system that classified sitting posture using a 3-axis accelerometer and Support Vector Machine (SVM). Ma et al. [15] proposed a cushion-based posture recognition system which was used to process pressure sensor signals for the detection of user’s posture in a wheelchair. References [16,17,18] also extracted human body features from a variety of sensors to estimate sitting postures. Although these methods can to a certain extent detect some unhealthy sitting postures, they have the limitations of placing the sensors in specific contact areas. They are susceptible to interference and have poor robustness. Besides, the inconvenience and sometimes even uncomfortable feelings that may occur when wearing the auxiliary equipment cause a poor user experience.

As for the vision-based methods, Song-Lin et al. [19] identified eight kinds of sitting postures based on face motion and skin color statistics by Principal Component Analysis (PCA). Mu et al. [20] extracted profile features through pattern matching based on Hausdorff distance and designed a surveillance system for human sitting postures. The related references [19,20,21] were sensitive to light and background because local facial information was not enough to express the characteristics of sitting postures. Once the face is obstructed, it is difficult to judge sitting posture from face information. Zhang et al. [22] applied 3D histograms of texture to action recognition. Wang et al. [23] combined image processing techniques with the depth images captured by a Kinect sensor to successfully recognize five distinct human postures. Yao et al. [24] proposed a new method of judging unhealthy sitting postures based on detecting the neck angle and the torso angle using a Kinect sensor. Tariq et al. [25] proposed that augmenting data from multiple sensing devices such as the Microsoft Kinect and smartwatches could significantly improve the detection performance once incorporated in the context of an IoT framework. Although the related references [23,24] could detect unhealthy sitting posture to a certain degree, these methods only focused on extracting features of humans themselves and lacked understanding of the relevancies among objects in the scene, so the existing methods could not perform effectively in complicated environments. Object detection and object tracking methods such as the method proposed by Zhang et al. [26] are essential to understand relevancies among objects in the scene. Some related literature [27,28] showed that sitting posture was not only relevant to the individual’s posture, but also relevant to the health constraint relationship between human and workplace scenario. Therefore, estimating sitting posture is the interactive result between humans and scenes in the workplace. The existing methods only extracted features of the trunk or contour. Some methods overlook relevant features from the scene. The appearance of this situation will inevitably lead to detection errors and missing detection for unhealthy sitting postures.

To alleviate these problems of the existing methods discussed above, a novel approach for unhealthy sitting posture detection during screen-reading based on scene recognition and semantic analysis is proposed in this paper. The key skeletal points of human body are detected and tracked with a Microsoft Kinect sensor. Meanwhile, a deep learning method is used in the scene recognition of our method to accurately detect objects and extract relevant features. Then our method performs semantic analysis through Gaussian-Mixture behavioral clustering for scene understanding. The relevant features in the scene and skeletal features in human are fused into semantic features to discriminate various types of sitting postures. The rest of this paper is organized as follows: Section 2 presents the framework of our proposed method. Scene recognition and semantic analysis in our proposed method are described in details in Section 3. Section 4 discusses our experiments and results. The paper is concluded in Section 5.

## 2. Framework of Our Proposed Method for Unhealthy Sitting Posture Detection

**Figure 1:**
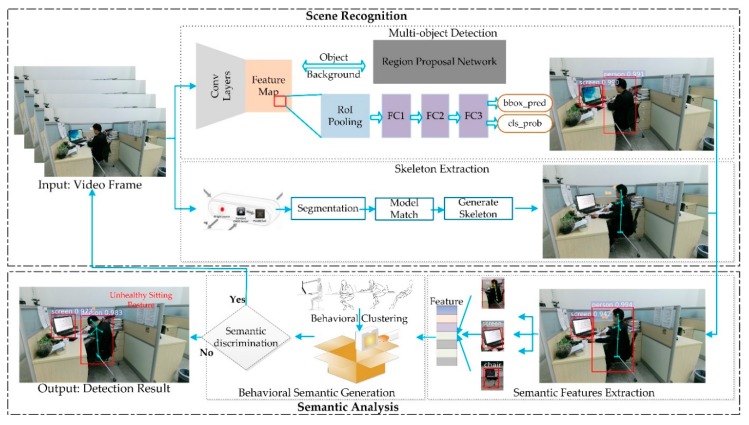
Framework of our proposed method for sitting posture detection. The framework is divided into two parts, i.e., the scene recognition and the semantic analysis.

The framework of our proposed method is illustrated in Figure 1. The method for unhealthy sitting posture detection mainly consists of two parts:

The first part is the scene recognition based on multi-object detection and skeleton extraction. In multi-object detection, a deep learning method, Faster Region-based Convolutional Neural Network (R-CNN), is used to extract the space and location information of the scene, i.e., person, screen and furniture. In the object detection network, the feature map is extracted based on a Visual Geometry Group network (VGGnet) [29], including 13 convolutional layers, three fully connected layers and four max pooling layers. A Region Proposal Network (RPN) is used to predict region proposals from the feature map. The main purpose of RPN is to classify objects and regress box coordinates. By using a sliding window, each feature point will generate the candidate regions of objects. Region of Interest pooling (RoI pooling) [30] are used to resize the object features to the same length. After the integration of the fully connected layer (i.e., FC1, FC2 and FC3 in Figure 1), these candidate boxes in the region proposal network are recognized by a classifier and located by regression. In skeleton extraction, the relevant features are calculated from the space and location information. Meanwhile, in order to obtain the features of human themselves, the key points of the human body are detected and tracked.

The second part is the semantic analysis based on behavioral semantic generation and behavioral clustering, which includes a semantic features extraction module and a semantic module. In the semantic feature extraction, a multi-dimensional feature (i.e., Feature in Figure 1) is generated by extracting the relevant features in the scene and the skeletal features extracted from humans. In behavioral semantic generation, Gaussian-Mixture Clustering is used to generate behavioral semantics. Based on the behavioral semantic clustering, the multi-dimensional features are fused into the semantic features to discriminate between various types of sitting postures.

## 3. Our Proposed Method Based on Scene Recognition and Semantic Analysis

### 3.1. Scene Recognition

The existing methods only focus on extracting features of human themselves and lack understanding of relevancies among objects in the scene. To alleviate these problems, our method firstly performs scene recognition to obtain abundant location and space information. The scene recognition consists of two parts, i.e., the multi-object detection using Faster R-CNN, and skeleton extraction using a Microsoft Kinect sensor.

#### 3.1.1. Multi-object Detection Using Faster R-CNN

Object detection in machine learning is a supervised classification or regression problem. More and more researchers are keen on multi-object detection based on deep learning models. Regression can output continuous coordinates and locate bounding-boxes, but using a model for multi-object detection is complicated and tedious. For object detection as classification, Convolutional Neural Network (CNN) obtains many different crops of the image generated by a sliding window, and then classifies each crop as object or background. Although these methods can achieve certain accuracy in object detection, the sliding window involves enormous parameters and causes complex operations. Therefore, the methods of object detection based on region proposal were designed by Girshick et al. [31]. As illustrated in Table 1, R-CNN [31], Spatial Pyramid Pooling Network (SPPnet) [32] and Fast R-CNN [33] replaced the sliding window with Selective Search. The Selective Search finds region proposals that are likely to contain objects by texture and color. It generates 1 k region proposals within approximately a second.

**Figure 2:**
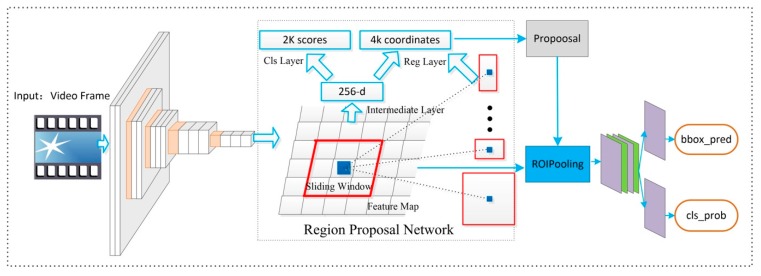
Our network of multi-object detection, include VGG16 and region proposal network.

The first generation method of object detection based on region proposal mainly comprises multiple convolutional nets and multiple classifiers which are made up of the Selective Search, CNNs and SVMs. The R-CNN needs to perform a forward CNN feature extraction for each proposal obtained from the Selective Search. Therefore, the amount of calculation is very large and cannot be completed in real-time. In addition, due to the existence of a fully connected layer, it is necessary to strictly ensure that the input proposal is eventually resized to the same scale size, which causes image distortion to a certain extent. To avoid the loss of image information caused by image distortion, in SPP-net, the original pool5 is replaced with a spatial pyramid pooling layer. This spatial pyramid pooling layer can make the length of feature vectors be the same and cannot request resizing of the original feature map. The training processing of R-CNN and SPPnet is similar. It is divided into multiple phases, so its implementation processing is more complicated. These two methods firstly use the Selective Search method to extract proposals, then use CNN to implement feature extraction. Finally, the classifier is trained based on a SVM algorithm. On this basis, the bounding box of the object can be further learned. Girshick [33] proposed Fast R-CNN which achieved multi-task and multi-scale end-to-end training. The model architecture of Fast R-CNN was consisted of share convolutional net and RoI pooling. As shown in Table 1, the time for generating region proposals takes most of the test time in the above four detection methods. The first step in the above four methods is to use the Selective Search method to extract the proposals in the image. The Selective Search based on CPU takes about 2 s for all proposals in an image. Without taking the proposal extraction into consideration, Fast R-CNN can basically perform target detection in 0.32 s per frame. However, if one considers from the perspective of end-to-end, it is clear that the proposal extraction becomes the bottleneck that affects the performance of the end-to-end method. Although the latest EdgeBox algorithm has improved the accuracy and efficiency of candidate frame extraction to a certain extent, it still takes 0.2 s to process an image. Therefore, Ren et al. [30] proposed a new Faster-R-CNN algorithm, which introduced the Region Proposal Network (RPN) to extract proposals. The RPN network is a full-convolutional neural network. Proposal extraction can be achieved by sharing the features of the convolutional layer, as shown in Figure 2. The RPN extraction of a proposal only requires 10 ms.

**Figure 3:**
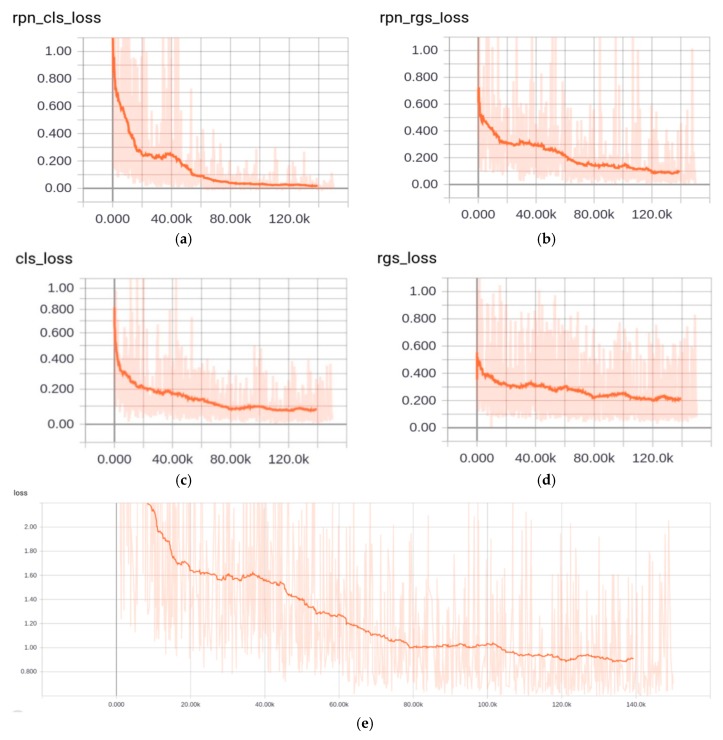
The curves of loss function in the processing of our training. (**a**) is the classification loss in RPN. (**b**) is the regression loss in RPN. (**c**) is the classification loss in object detection. (**d**) is the regression loss in objection. (**e**) is the overall loss which is the total of the above four losses.

The excellent algorithm Faster R-CNN is applied in multi-object detection in this paper. Our multi-object detection network is shown in Figure 2. The network is made up of three parts. There are many convolutional neural networks in the related literature [29,34,35]. The first part is feature map extraction based on Visual Geometry Group network (VGGnet) [29], including 13 convolutional layers, three fully connected layers and four max pooling layers. The second part is a region proposal network (RPN) to predict region proposals from feature map. By the sliding window, each feature point will generate nine anchors (three scales and three ratios). RPN mainly classifies object or not object and regresses box coordinates. Region of Interest pooling (RoI pooling) [30] is used to resize the object features to same length. After the integration of a fully connected layer (i.e., FC1, FC2 and FC3 in Figure 1), these candidate boxes in the region proposal network are recognized by a classifier and located by regression. As for RPN, on the convolutional feature map of conv5-3, an n × n sliding window (setting n = 3 in our implementation, i.e., using a 3 × 3 sliding window) is used to generate a full-length feature of 256 dimensions (256d) (corresponding to ZF network) or 512 dimensions (512d) (corresponding to VGGnet). After generating the 256-dimensional or 512-dimensional feature, a full-connected layer of two branches is generated. The first branch *reg-layer* is used to predict the coordinates of the corresponding anchor of the proposal’s central anchor, *x*, y, and width, height, *w*, h. The second branch *cls-layer* is used to determine if the proposal is foreground or background. The handling of the sliding windows ensures that *reg-layer* and *cls-layer* are associated with the full feature space of conv5-3. The loss functions of RPN and object detection are all recorded in Figure 3. We adopt the following loss function defined in [30]:

(1) $$\;L\left(\left\{p_{i}\right\},\left\{t_{i}\right\}\right)=\frac{1}{N_{cls}}\underset{i}{\sum }L_{cls}\left(p_{i},p_{i}^{*}\right)+\lambda \frac{1}{N_{reg}}\underset{i}{\sum }p_{i}^{*}L_{reg}\left(t_{i},t_{i}^{*}\right)$$

- i is the index of an anchor in a mini-batch.
- $p_{i}$ is the predicted probability of anchor i being an object.
- $p_{i}^{*}$ is the ground-truth label, whose value is 1 if the anchor is positive and 0 if the anchor is negative.
- *t_i_* is a vector representing the 4 parameterized coordinates of the predicted bounding box.
- $t_{i}^{*}$ is the ground-truth box associated with a positive anchor.
- The classification loss $L_{cls}$ is log loss over two classes (object versus not object).
- The regression loss $L_{reg}=\left(t_{i},t_{i}^{*}\right)=R\left(t_{i}-t_{i}^{*}\right)$ where R is the robust loss function (smooth $L_{1}$).
- The outputs of the *cls* and *reg* layers consist of $\left\{p_{i}\right\}\;$ and $\left\{t_{i}\right\}$ respectively.
- λ is weighted by a balancing parameter.
- The mini-batch size (i.e., $N_{cls}$ = 256) and the reg term is normalized by the number of anchor locations (i.e., $N_{reg}$ = 2400). By default we set λ = 10, thus both *cls* and *reg* terms are roughly equally weighted.

(2) $$\begin{array}{c} t_{x}=\left(x-x_{a}\right)/\omega_{a},\;t_{y}=\left(y-y_{a}\right)/h_{a}\\ t_{w}=log\left(\omega /\omega_{a}\right),\;t_{h}=log\left(h/h_{a}\right)\\ t_{x}^{*}=\left(x^{*}-x_{a}\right)/\omega_{a},\;t_{y}^{*}=\left(y^{*}-y_{a}\right)/h_{a}\\ t_{w}^{*}=log\left(\omega^{*}/\omega_{a}\right),\;t_{h}^{*}=log\left(h^{*}/h_{a}\right)\; \end{array}$$

where x, y, w, h denote the box’s center coordinates including its widths and height. Variables $x,\;x_{a},\;x^{*}$ are for the predicted box, anchor box and ground truth box, respectively.

PASCAL VOC-2007 [36] is a standard dataset for measuring image classification and recognition capabilities. Everingham and others have provided images and annotations. It includes twenty object classes such as chair, dining table, person, sofa, screen. There are in total 9963 images in this dataset. Only 5362 of the images contain persons, chairs or screens. These images are selected to form a sub-dataset for training and testing our model. In our experiments, the ratio of image numbers of the training set to the test set from the sub-dataset is about 5:1. In other words, 4500 images in the dataset are used as the training set and 862 images are used as the testing set. 

After 150,000 iterations, the curves of loss function in the processing of our training are recorded in Figure 3. Figure 3a is the classification loss in RPN. Figure 3b is the regression loss in RPN. Figure 3c is the classification loss in object detection. Figure 3d is the regression loss in objection. Figure 3e is the overall loss which is the total of the above four losses. The magnitude of mean Average Precision (mAP) is 70.8% on testing set. Our model has been also tested in our self-collected video frame. According to the changes of the loss value in Figure 3a–e and the magnitude of mAP on testing set, these curves prove that the above five loss functions have good convergence.

After 150,000 iterations, the results of recall, precision and accuracy of the algorithm for persons, chairs and screens are shown in Table 2. TP means true positive, TN means true negative, FP means false positive and FN means false negative. We have  recall=TP/(TP+FN), precison=TP/(TP+FP) and  accuracy=(TP+TN)/(TP+TN+FP+FN). Table 2 demonstrates that Faster-RCNN can detect the objects effectively. The accurate information of locations and objects can be extracted based on Faster R-CNN to analyze scene.

#### 3.1.2. Skeleton Extraction Using Microsoft Kinect Sensor

The human skeleton information is extracted from the depth images. Kinect [37] can decode the infrared-coded light and can generate an image that can represent the surrounding environment with three-dimensional depth information by means of Complementary Metal Oxide Semiconductor (CMOS) infrared sensors. The sensor can generate a depth-of-field image stream at a speed of 30 frames per second and reproduce the 3D surroundings in real time. After the depth image is obtained, the key points of the human body are detected and tracked. The skeleton tracking system is used to search for moving objects that may be human in the depth image. In order to shorten the response time, Kinect uses a segmentation strategy to optimize preprocessing to distinguish the human body from background environment. In the following processing flow, only the portion of the split mask is transmitted to reduce the amount of motion calculation. Next, a pixel level evaluation is performed to distinguish different parts of the human body. A skeleton structure diagram of the human body is obtained based on the tracked joint points. Kinect can assess the actual position of the human body most accurately based on sufficient information. 

**Figure 4:**
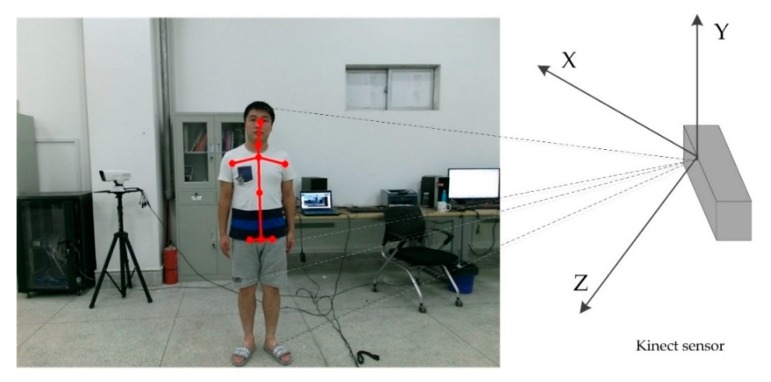
Skeleton extraction using Microsoft Kinect sensor in our experiments.

In this paper, the health constraint relationship between joints is considered, so the experiment of sitting posture detection mainly extracts the head joints, neck joints, and lumbar joints of the human head (as shown in Figure 4). We only need to obtain the position information of the upper body skeletal points. Even if the data of the lower body skeletal points is not stable or does not exist, it will not have a bad influence on the upper body skeletal data. Thus, our method solves the problem that the user cannot be recognized by Kinect directly when sitting in the chair.

### 3.2. Semantic Analysis

#### 3.2.1. The Definition of Healthy Sitting Posture

**Figure 5:**
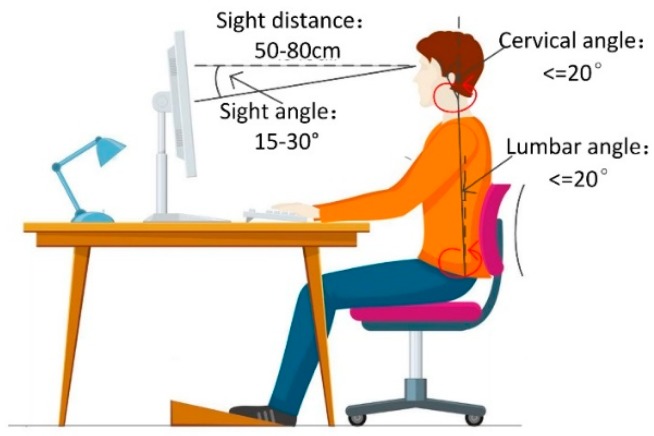
The definition of healthy sitting posture based on ergonomics.

There are lots of related references discussing what kind of standards are considered as healthy sitting postures. McAtamney et al. [38] proposed that the lumbar spine angle and the cervical spine angle greater than 20° were judged as unhealthy sitting postures. Burgess-Limerick et al. [39] stated a healthy distance of eye and human was about 40–70 cm. Springer [40] et al. showed that the best angle for visual screen was 15°–30° below horizontal sight. Based on ergonomics [37,41], our method comprehensively extracts features that are strongly correlated with sitting posture health from human body joints, persons and scenes [42], as illustrated in Figure 5.

#### 3.2.2. Semantic Feature Calculation

As we stated above, the existing methods only focus on extracting features of humans themselves and lack understanding of the relevancies among objects in the scene. It is necessary for us to extract more useful features to judge sitting posture. In the sitting posture detection, the objects in the scene are mainly composed of persons, chairs and screens. Not only do we focus on the characteristics of persons, but also relevant features are extracted to measure the relationship between the objects. By these relevant semantic features, our method not only accurately and effectively identified varieties of unhealthy sitting postures in screen-reading but also discriminated unhealthy sitting postures such as unhealthy sitting postures caused by sight distance and sight angle, while the existing methods cannot handle these.

**Figure 6:**
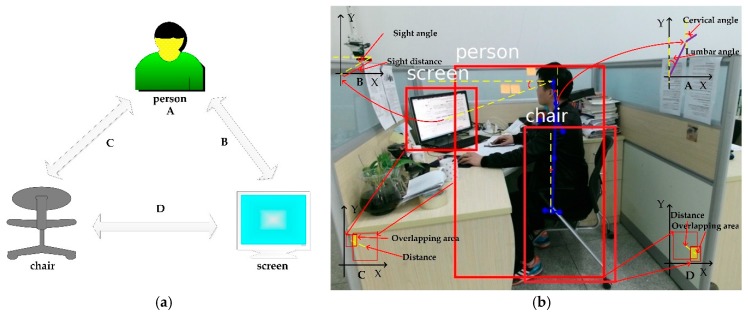
The diagram of semantic analysis. (**a**) The diagram of relevant semantic features; (**b**) The extraction method of semantic features A, B, C and D. A is extracted from human skeleton joints. B is extracted from person and screen. C is extracted from person and chair. D is extracted from chair and screen.

As illustrated in Figure 6a, these semantic features are defined as A, B, C, and D. According to Figure 6b, semantic feature A extracted from human skeletal points is the main parameter to measure unhealthy sitting posture, including lumbar angle and cervical angle. Semantic feature B extracted from person and screen contains sight angle and sight distance. Semantic feature C and D are extracted from objects in the scene, spatial relationships are represented by overlapping area and spatial distance.

**Figure 7:**
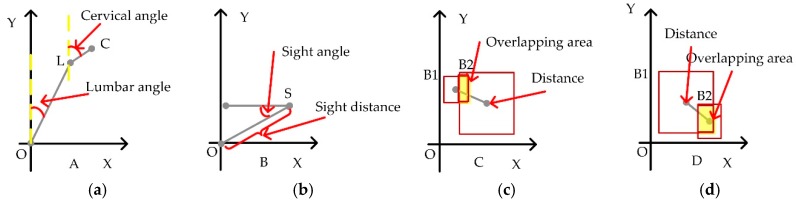
The calculation of semantic features. (**a**–**d**) correspond to the calculation of semantic features A, B, C and D.

In Figure 7a, semantic feature A, the direction vector in the vertical direction is denoted as $\vec{e_{y}}$. The lumbar angle (defined as *α*) and cervical angle (defined as *β*) are calculated by the following Equations (3) and (4):

(3) $$\;\alpha =acos\left\{\;\frac{\vec{OL}\cdot \;\vec{e_{y}}}{\left|\vec{OL}\right|\left|\vec{e_{y}}\right|}\;\right\}\;$$

(4) $$\;\beta =acos\left\{\;\frac{\vec{LC}\cdot \;\vec{e_{y}}}{\left|\vec{LC}\right|\left|\vec{e_{y}}\right|}\;\right\}\;$$

In Figure 7b, semantic feature B, the direction vector in the horizontal direction is denoted as $\;\vec{e_{x}}$. The sight angle (denoted as *γ*) and sight distance (denoted as  δ) are calculated by the following Equations (5) and (6):

(5) $$\;\gamma =acos\left\{\;\frac{-\vec{SO}\cdot \;\vec{e_{x}}}{\left|\vec{SO}\right|\left|\vec{e_{x}}\right|}\;\right\}\;$$

(6) $$\;\delta =\left|\vec{SO}\right|$$

Semantic feature C and semantic feature D are illustrated in Figure 7c,d. The distance between objects and the overlapping area between objects are extracted to judge spatial relationship in the screen-reading scene. Semantic features C and D mainly assist in identifying sitting posture and determining whether the sitting posture is healthy or not. The coordinates of B are defined as (*x*, *y*, *w*, *h*), where (*x*, *y*) is the upper left coordinate of rectangle B, and *w* is the width of rectangle B, and *h* is the height of rectangle B. The distance (denoted as *d*) and overlapping area (denoted as *Oa*) are calculated by following Equations (7) and (8):

(7) $$\;d=\;\sqrt{{\left[\left(x_{1}+\;\frac{w_{1}}{2}\right)-\left(x_{2}+\;\frac{w_{2}}{2}\right)\right]}^{2}+{\left[\left(y_{1}-\;\frac{h_{1}}{2}\right)-\left(y_{2}-\;\frac{h_{2}}{2}\right)\right]}^{2}}\;$$

(8) $$\;Oa=\;\left|\max\left(x_{1},\;x_{2}\right)-\min\left(x_{1}+w_{1},x_{2}+w_{2}\right)\right|\cdot \left|\min\left(y_{1},\;y_{2}\right)-\max\left(y_{1}-h_{1},y_{2}-h_{2}\right)\right|\;$$

where ($x_{1}$,$y_{1}$,$w_{1}$,$h_{1}$) is the coordinates of rectangle B1, and ($x_{2}$,$y_{2}$,$w_{2}$,$h_{2}$) is the coordinates of rectangle B2.

**Figure 8:**
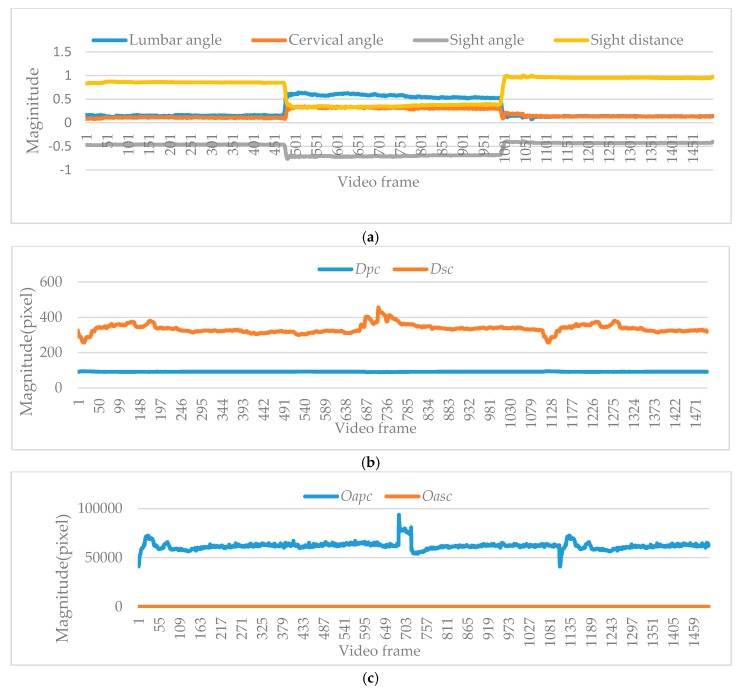
The semantic feature curves. (**a**) The semantic feature curves of lumbar angle, cervical angle, sight angle and sight distance. (**b**) The semantic feature curves of distance between person and chair, distance between screen and chair. (**c**) The semantic feature curves of overlapping area between person and chair, overlapping area between screen and chair.

As illustrated in Figure 8a–c, all semantic features discussed by us are extracted, including lumbar angle α, cervical angle *β*, sight angle *γ*, sight distance *δ*. The distances between person and chair and distance between screen and chair are defined as *Dpc* and *Dsc*. Overlapping area between person and chair and overlapping area between screen and chair are defined as *Oapc* and *Oasc*, respectively.

From the 1st frame to 476th frame, a person read the screen and maintained a healthy sitting posture. The magnitude of the lumbar angle is about 0.15 (8.59°). The magnitude of the cervical angle is about 0.11 (5.72°). The magnitude of the sight angle is about −0.46 (−24.36°). The magnitude of the sight distance is about 0.86 m. The cervical angle and lumbar angle are less than 20°. According to the healthy sitting posture defined in Figure 5, the behavior from 1st frame to 476th frame is judged as a healthy sitting posture.

From the 477th frame to 1001st frame, the human body is tilted forward to read screen and kept an unhealthy sitting posture. The magnitude of the lumbar angle is about 0.58 (33.23°). The magnitude of the cervical angle is about 0.36 (20.63°). The magnitude of the sight angle is about −0.69 (−39.54°). The magnitude of the sight distance is about 0.37 m. According to the healthy sitting posture defined in Figure 5, the behavior from the 477th frame to 1001st frame is judged as an unhealthy sitting posture.

As shown in Figure 8b,c, semantic feature C and semantic D (i.e., the overlapping area and the spatial distance between objects in the scene) are also proposed in our paper to extract relevant features in the scene. These features are mainly to identify the occurrence of sitting behavior in the scene. When people use the computer in front of the screen, there is always a spatial relationship between related objects such as people, screens, and chairs. These parameters in semantic feature C and semantic feature D are used to determine these spatial relationships. According to Figure 8b,c, when people are sitting and using the computer, the values of spatial distance and the overlapping area are all stable. When a person stands near the char, walks near the chair or moves away from the chair suddenly, these two parameters will be changed and used to judge whether the subject is sitting in the chair or not. These parameters can be used to identify the sitting posture and then detect the unhealthy sitting posture.

#### 3.2.3. Semantic Generation using Gaussian-Mixture Clustering

After abundant semantic features are obtained, a Gaussian-Mixture model are applied in generating behavioral semantic clustering. Suppose we have observation features $\;\left\{x_{1},\;x_{n},\;\ldots ,\;x_{m}\right\}$, these features are classified by mixture of Gaussian functions. The mixing coefficients, the mean vector and covariance vector are represented by $\;\alpha_{i},\;\mu_{i},\;\Sigma_{i}$. The probability density distribution function of features is written in the form:

(9) $$\;p(x|\;\mu_{i},\;\Sigma_{i})=\;\frac{1}{{\left(2\pi \right)}^{\;\frac{n}{2}\;}{\left|\Sigma \right|}^{\;\frac{1}{2}}}\;e^{\;-\frac{1}{2}\;{\left(x\;-\;\mu \right)}^{\;T}{\;\Sigma }^{-1}\;\left(x\;-\;\mu \right)}\;$$

The Gaussian-Mixture distribution can be written as a linear superposition of Gaussians:

(10) $$\;p_{M}\left(x\right)=\;\overset{k}{\underset{i=1}{\sum }}\alpha_{i}\cdot p(x|\;\mu_{i},\;\Sigma_{i})\;$$

Here we introduce a *k*-dimensional binary variable  z that has a 1-of-*k* representation. For a particular element $\;z_{i}$, its value satisfies $\;1\geq \alpha_{i}\geq 0\;\mathrm{and}\;\overset{k}{\underset{i=1}{\sum }}\alpha_{i}=1$. If *k* = *j*, then $\;P\left(z_{j}=i\right)=\;\alpha_{i}$. Next, we introduce the conditional probability of *z* given *x*. $\lambda_{ji}$ is used to denote $p_{M}\left(z_{i}=i\;|\;x_{i}\right)$, whose value can be found using the following Bayes’ theorem:

(11) $$\begin{array}{c} \;\lambda_{ji}=\;p_{M}\left(z_{i}=i\;|\;x_{i}\right)=\;\frac{P\left(z_{j}=i\right)\cdot p_{M}\left(x_{j}\;|\;z_{j}=i\right)}{p_{M}\left(x_{j}\right)}\\ \;=\;\frac{\alpha_{i}\cdot p(x_{j}\;|\;\mu_{i},\;\Sigma_{i})}{\sum_{i=1}^{k}\alpha_{i}\cdot p(x\;|\;\mu_{i},\;\Sigma_{i})}\; \end{array}$$

Now, we need find out the model parameters $\;\left\{\left(\alpha_{i},\;\mu_{i},\;\Sigma_{i}\right)|1\leq i\;\leq k\right\}$ to make maximum posterior probability of $\;\lambda_{ji}$ once we observed *x*:

(12) $$\lambda_{j}=\underset{i\;\epsilon \;\left\{1,\;2,\ldots ,k\right\}}{\arg\;\max}\lambda_{ji}\;$$

In order to optimize Equation (12), we use the following log of likelihood function:

(13) $$\;LL\left(D\right)=ln\left(\overset{m}{\underset{j=1}{\prod }}p_{M}\left(x_{j}\right)\right)\;=\;\overset{m}{\underset{j=1}{\sum }}ln\left(\overset{k}{\underset{i=1}{\sum }}\alpha_{i}\cdot p(x_{j}|\;\mu_{i},\;\Sigma_{i})\right)\;$$

If we set the derivatives of  LL(D) in Equation (13) with respect of the means $\;\mu_{i}$ of the Gaussian components to zero, we obtain:

(14) $$\;\frac{\partial LL\left(D\right)}{\partial \mu_{i}}=\;\overset{m}{\underset{j=1}{\sum }}\frac{1}{\sum_{l=1}^{k}\alpha_{l}\cdot p(x_{j}|\;\mu_{l},\;\Sigma_{l})}\cdot \frac{\partial \left(\alpha_{i}\cdot p(x_{j}|\;\mu_{i},\;\Sigma_{i})\right)}{\partial \mu_{i}}\;\;=\;\overset{m}{\underset{j=1}{\sum }}\frac{\alpha_{i}\cdot p(x_{j}|\;\mu_{i},\;\Sigma_{i})}{\sum_{l=1}^{k}\alpha_{l}\cdot p(x_{j}|\;\mu_{l},\;\Sigma_{l})}\;\left(x_{j}-\;\mu_{i}\right)=\;\overset{m}{\underset{j=1}{\sum }}\lambda_{ji}\;\left(x_{j}-\;\mu_{i}\right)=0\;$$

By solving for $\mu_{i}$ in Equation (14), we obtain:

(15) $$\;\mu_{i}=\;\frac{\sum_{j=1}^{m}\lambda_{ji}x_{j}}{\sum_{j=1}^{m}\lambda_{ji}}\;$$

Similarly, by setting the derivatives of $\;\frac{\partial LL\left(D\right)}{\partial \Sigma_{i}}=0$ and then solving for $\;\Sigma_{i}$, we obtain:

(16) $$\;\Sigma_{i}=\;\frac{\sum_{j=1}^{m}\lambda_{ji}{\left(x_{j}\;-\mu_{i}\right)}^{\;T}{\;\Sigma }^{-1}\;\left(x_{j}\;-\;\mu_{i}\right)}{\sum_{j=1}^{m}\lambda_{ji}}\;$$

As for the parameter of $\;\alpha_{i}$, the loss is defined as:

(17) $$L_{\alpha }\left(D\right)=LL\left(D\right)+\;m\left(\overset{k}{\underset{i=1}{\sum }}\alpha_{i}-1\right)\;$$

Similarly, we set the derivatives $\;\frac{\partial LL\left(D\right)}{\partial \alpha_{i}}=0$ as follows:

(18) $$\begin{array}{c} \;\frac{\partial LL_{\alpha }\left(D\right)}{\partial \alpha_{i}}=\;\overset{m}{\underset{j=1}{\sum }}\frac{1}{\sum_{l=1}^{k}\alpha_{l}\cdot p(x_{j}|\;\mu_{l},\;\Sigma_{l})}\cdot \frac{\partial \left(\alpha_{i}\cdot p(x_{j}|\;\mu_{i},\;\Sigma_{i})\right)}{\partial \alpha_{i}}+\;\frac{\partial \;m\left(\sum_{i=1}^{k}\alpha_{i}-1\right)}{\partial \alpha_{i}}\\ \;=\;\overset{m}{\underset{j=1}{\sum }}\frac{\alpha_{i}\cdot p(x_{j}|\;\mu_{i},\;\Sigma_{i})}{\sum_{l=1}^{k}\alpha_{l}\cdot p(x_{j}|\;\mu_{l},\;\Sigma_{l})}\;+m=\;\frac{1}{\alpha_{i}}\left(\overset{m}{\underset{j=1}{\sum }}\frac{\alpha_{i}\cdot p(x_{j}|\;\mu_{i},\;\Sigma_{i})}{\sum_{l=1}^{k}\alpha_{l}\cdot p(x_{j}|\;\mu_{l},\;\Sigma_{l})}\;+m\alpha_{i}\right)\;\\ \;=\;\frac{1}{\alpha_{i}}\left(\overset{m}{\underset{j=1}{\sum }}\lambda_{ji}\;+m\alpha_{i}\right)=0\; \end{array}$$

By solving for $\;\alpha_{i}$, we obtain $\alpha_{i}=\;\frac{1}{m}\;\overset{m}{\underset{j=1}{\sum }}\lambda_{ji}\;$.

Given a Gaussian-Mixture model, the goal is to maximize the likelihood function with respect to the parameters. An elegant powerful method for finding the maximum likelihood solution for models with latent variables is called the Expectation-Maximization algorithm, or EM algorithm [7]. Some initial magnitudes for means $\;\mu_{i}$, covariances $\;\Sigma_{i}$, and mixing coefficients $\;\alpha_{i}$ are selected by us. Then three iterations are alternated to generate our semantic clustering. In first iteration, the current values for the parameters are used to elevate the posterior probabilities $\;\lambda_{ji}$. In the second iteration, the probabilities in the first iteration are used to estimate the means $\;\mu_{i}$, covariances $\;\Sigma_{i}$, and mixing coefficients $\;\alpha_{i}$. In the third iteration, according to $\;\lambda_{j}=\underset{i\;\epsilon \;\left\{1,\;2,\ldots ,k\right\}}{\arg\;\max}\lambda_{ji}$, behavioral semantic clustering is generated, as shown in Algorithm 1.

**Algorithm 1.** The processing of generating behavioral semantic clustering.Input: semantic features $F=\;\left\{f_{1},f_{2},\ldots ,f_{m}\;\right\}$
   The number of category k
Output: $S\;=\;\left\{\;S_{1},S_{2},\ldots ,S_{k}\;\right\}.$
Repeat  for j = 1, 2, …, m do {    According to, calculate the posterior probability of $f_{j}:$
$\lambda_{ji}=\;p_{M}\left(z_{i}=i\;|\;f_{i}\right)$}  for i = 1, 2, …, m do {      Calculate mean vector: $\mu_{i}=\;\frac{\sum_{j=1}^{m}\lambda_{ji}f_{j}}{\sum_{j=1}^{m}\lambda_{ji}}$
      Calculate covariance matrix: $\Sigma_{i}=\;\frac{\sum_{j=1}^{m}\lambda_{ji}{\left(f_{j}\;-\mu_{i}\right)}^{\;T}{\;\Sigma }^{-1}\;\left(f_{j}\;-\;\mu_{i}\right)}{\sum_{j=1}^{m}\lambda_{ji}}$
      Calculate mixture coefficient: $\alpha_{i}=\;\frac{1}{m}\;\overset{m}{\underset{j=1}{\sum }}\lambda_{ji}\;$}  Update semantic parameters $\left\{\;\left(\;\alpha_{i},\;\mu_{i},\;\Sigma_{i}\;\right)\;|\;1\leq i\;\leq k\right\}$
Until find out the optimization of $\lambda_{j}$
  for j = 1, 2, …, m do {    According to $\lambda_{j}=\underset{i\;\epsilon \;\left\{1,\;2,\ldots ,k\right\}}{\arg\;\max}\lambda_{ji}$, generate behavioral semantic clustering:
$S=S_{\lambda_{j}}\cup \{f_{j}\}$
  }

#### 3.2.4. Semantic Discrimination

**Figure 9:**
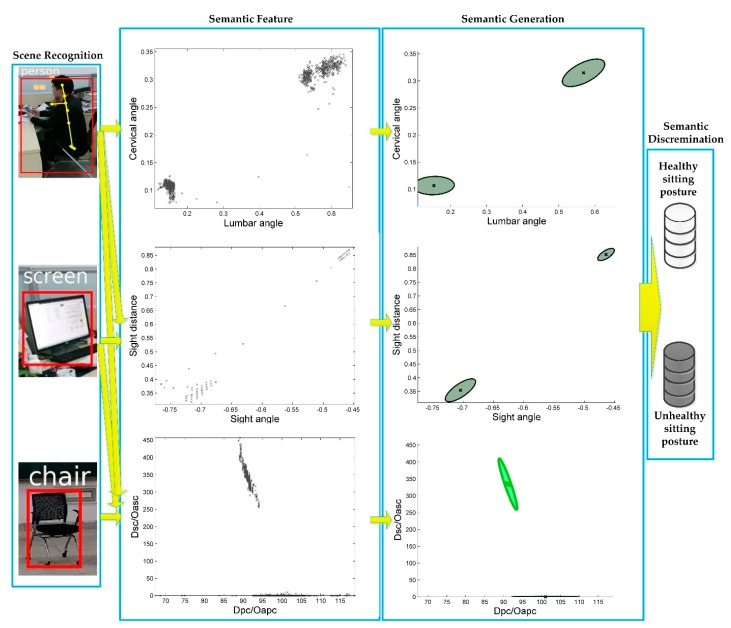
The processing of semantic analysis (i.e., scene recognition, semantic feature extraction and semantic generation) are illustrated for semantic discrimination.

The four steps of semantic analysis are illustrated in Figure 9.

*First Step: scene recognition*. In Figure 8, people sit in the chair and read screen. This scene is well recognized by Faster R-CNN. Persons, screens, chairs, and other furniture are recognized correctly. At the same time, human skeleton is also detected and tracked by the Kinect sensor. These tasks are necessary and preconditions for the semantic feature extraction.

*Second step: semantic feature extraction*. In this step, the distribution of semantic feature A (including lumbar and cervical angles) is extracted from the human skeleton. The distribution of semantic feature B (including sight distance and sight angle) is extracted based on the human and screen positions. The distributions of semantic feature C and D (including *Dpc*, *Dsc*, *Oapc* and *Oasc*) are extracted from the relevancy in the scene, i.e., person, screen and chair.

*Third step: semantic generation*. Our experiments are based on the derivation of formulas in Section 3.2.3 and Algorithm 1. As the experimental results in Figure 9 demonstrate, the distribution of semantic features can be well clustered, and feature magnitudes of healthy and unhealthy sitting postures are all in the corresponding areas, so we can conclude that all areas are easily distinguished by our method.

*Fourth step: semantic discrimination*. According to the semantic generation result using Gaussian-Mixture clustering, the corresponding semantic feature clustering region are put into corresponding databases (including healthy sitting posture database and unhealthy sitting posture database).

## 4. Result

### 4.1. Self-Collected Test Dataset and Some Detection Results

**Figure 10:**
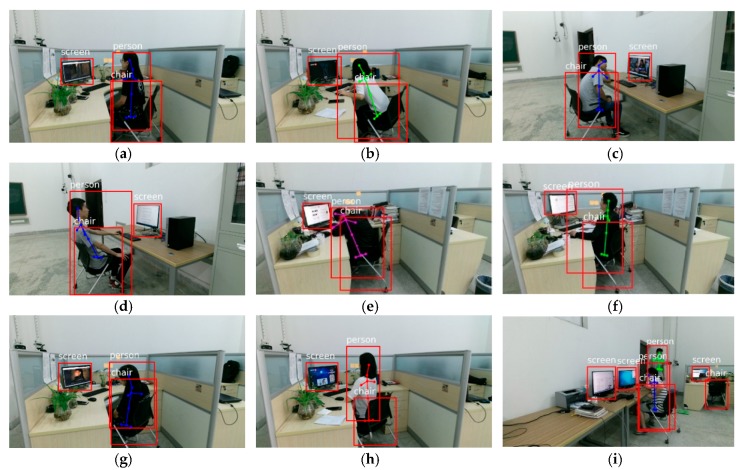
Some detecting results based on self-collected dataset by our proposed method. (**a**) example of healthy sitting posture in screen-reading. (**b**–**i**) are representative unhealthy sitting postures in screen-reading detected by our proposed method.

Detecting unhealthy sitting posture accurately is our main purpose. In order to prove that our method has better performance than other methods, our method must be tested against a standard dataset. Due to lack suitable standard datasets for sitting posture measurement, we collected our own test dataset. Our self-collected dataset is collected by a Microsoft Kinect 2.0 which is fixed in 1.4 m above the ground. The distance between camera and scene is about 2~3 m. Person, screen and chair are included in our video samples. All videos are composed of RBG and depth information. Seven kinds of common unhealthy sitting postures and a variety of healthy sitting postures which may appear in screen-reading are contained in our self-collected dataset. Each unhealthy sitting posture contains about 60~65 videos. The duration of each video is about 20~90 s and each video contains 600~2700 frames. Our dataset contains eight persons in different scenes, including eight boys and four girls (aged between 20~30 years old and height between 1.5~1.8 m). There are total 500 sample videos in our self-collected dataset. In order to more visually express our self-collected dataset, nine representative detecting results are randomly selected in the experiment, as shown in Figure 10. Our main purpose is the detection of unhealthy sitting posture of people in screen-reading such as programmers and office worker, so there must be these predefined objects existing in the scene.

In order to describe different sitting postures better, different types of sitting postures in screen-reading (i.e., Figure 10a–i in self-collected dataset) are introduced in details as follows.

- Healthy sitting posture as shown in Figure 10a. The lumbar angle and cervical angle are less than 20°. The sight distance is about 80 cm. The sight angle is in 15°~30°.
- Leaning forward as shown in Figure 10b. When a person reaches out to read the screen, leaning forward causes an excessive lumbar angle, cervical angle and a small sight distance.
- Holding the head as shown in Figure 10c. When a person holds his head with a hand on the desk, the leaning body results in unhealthy sitting posture.
- Leaning backward as shown in Figure 10d. When a person leans on the chair, the excess lumbar angle, cervical angle and sight distance cause an unhealthy sitting posture.
- Bent over as shown in Figure 10e. When a person squats on the desk, the excessive bending angle results in an unhealthy sitting posture.
- Looking up as shown in Figure 10f. Because the location of the screen is too high or the chair is low, looking up at screen causes an unhealthy sitting posture.
- Body sideways as shown in Figure 10g. When a person leans on the side of the chair, the excessive lumbar angle and cervical angle cause an unhealthy sitting posture.
- Small sight distance as shown in Figure 10h. Here the eyes are too close to the screen.
- Holding the head in a complicated environment as shown in Figure 10i. A person is holding his head with a hand on the desk, and the scene contains multiple objects (i.e., multiple persons, multiple screens and multiple chairs).

### 4.2. Quantitative Analysis

Quantitative analysis is first performed in our experiment to verify the robustness of our proposed method. As discussed above, a scene recognition and semantic analysis approach for detecting unhealthy sitting postures while screen-reading is proposed in this paper. The existing methods only focus on extracting the features of human themselves and lack understanding of relevancies among objects in the scene. Since the auxiliary-equipment-based methods and the existing wearing-equipment-based methods used sensors to collect values such as pressure and speed and then determined the unhealthy sitting postures, the comparison performance test using these sensors could not be accomplished with our self-collected dataset. As for the vision-based methods such as those using face motion and skin color statistic to detect sitting posture, performance comparison tests could not be conducted either because of a lack of visual information viewed from the front. Our dataset was collected from a side view. Therefore, the wearing-equipment-based methods and the vision-based methods cannot be compared with our proposed method on the same dataset. To summarize the above, it is impossible to conduct the comparison experiments with the existing wearing-equipment-based methods and the vision-based methods on the same dataset.

To quantitatively demonstrate better performance of our proposed method, comparison experiments using our collected dataset are conducted against the Kinect-based method described in [24] which judged unhealthy sitting posture based on the neck angle and the torso angle. The comparison results are shown in Table 3. 

The compared method is a Kinect-based method, i.e., judging unhealthy sitting posture based on the neck angle and the torso angle [24]. The results in Table 3 demonstrate the following conclusions: This method only depends on neck angle and torso angle. Therefore, this method has poor robustness to some special sitting postures, i.e., unhealthy sitting postures caused by sight angle and sight distance. As for unhealthy sitting postures caused by too small sight distance, 54 of 65 sample videos are detected as unhealthy sitting posturea. Similarly, this method only focuses on extracting features of human themselves and lacka understanding of relevancies among objects in the scene. There have been more false healthy sitting posture detections and missed unhealthy sitting posture detections.

In order to evaluate the overall performance of our method, the detection results of the Kinect-based method are counted based on Table 3, and illustrated in Table 4. True Positive (*TP*) means unhealthy samples judged as unhealthy sitting postures. True Negative (*TN*) means the healthy samples judged as healthy sitting postures. False Positive (*FP*) means the healthy samples judged as unhealthy sitting postures. False Negative (*FN*) means the unhealthy samples judged as healthy sitting postures. The highest *TP* and *TN* values in our proposed method demonstrate the reliability of our proposed method in detecting unhealthy sitting posture, the lowest *FP* and *FN* values demonstrate our proposed method has lower error detection and missing detection than the Kinect-based method.

**Figure 11:**
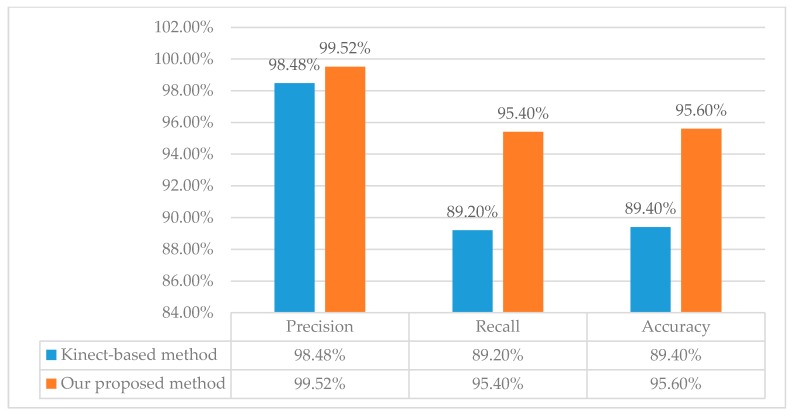
Comparison of our method and the Kinect-based method about precision, recall and accuracy based on self-collected dataset.

Besides, the parameter precision, recall and accuracy are also verified by our statistics to prove the reliability and accuracy of our proposed method, as illustrated in Figure 11. We have  Precision=TP/(TP+FP), Recall=TP/(TP+FN), and  Accuracy=(TP+TN)/(TP+TN+FP+FN). Obviously, our method has higher precision, recall and accuracy than the Kinect-based method.

Unhealthy sitting postures in complicated scenes are also tested. As stated in Section 3.2.2, eight different parameters (i.e., lumbar angle, cervical angle, sight angle, sight distance, distance between person and chair, distance between screen and chair, overlapping area between person and chair, overlapping area between screen and chair) are extracted in our proposed method. The first four of eight parameters (i.e., lumbar angle, cervical angle, sight angle, sight distance) are used to measure whether the sitting posture is healthy or not, while the last four of eight parameters (i.e., distance between person and chair, distance between screen and chair, overlapping area between person and chair, overlapping area between screen and chair) are used to understand relevancies among objects in the scene.

**Figure 12:**
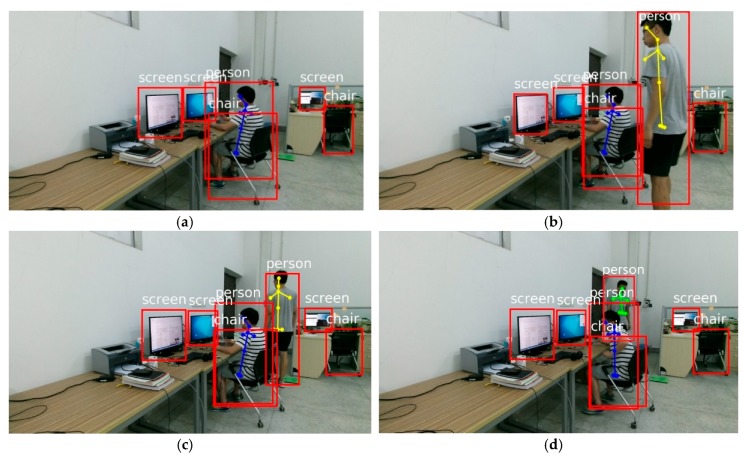
Unhealthy Sitting postures in complicated scenes are detected by our proposed method. In (**a**–**c**), when human body bends over and sitting on a chair, excessive cervical angle and excessive lumbar angle cause unhealthy sitting postures. In (**d**), when a person holds their head with hand on the desk, the distorted body results in unhealthy sitting posture.

The unhealthy sitting postures in Figure 12 are detected by our proposed method. In Figure 12a–c, a person is bending over and sits on the chair, so the oversized lumbar angle and cervical angle cause an unhealthy sitting posture. Our proposed method can determine the relationship between persons and objects in complicated environments. The first complicated environment is shown in Figure 12a. When a person is working with a screen, there are two extra screens next to him in the workplace. According to the last four parameters (i.e., distance between person and chair, distance between screen and chair, overlapping area between person and chair, overlapping area between screen and chair), the relationship between the person and corresponding screen can be well determined, and the extra screens will be excluded. Then the first four parameters can be calculated correctly (i.e., lumbar angle, cervical angle, sight angle, sight distance) to determine whether this sitting posture is healthy or not. Secondly, multiple persons, multiple screens and multiple chairs are arranged in Figure 12b–d. This situation cannot be handled by traditional methods. Our proposed method can exclude extra persons and other objects in complicated environments so that unhealthy sitting postures can be effectively detected. Experimental results demonstrated that our method accurately and effectively detected various types of unhealthy sitting postures in screen-reading and avoided error detection in complicated environments.

In the above experiments, dramatic changes within five seconds (150 frames) will not be judged as an unhealthy sitting posture in our implementation. When subjects suddenly move from the chair, all features will change for a short time. Only stationary unhealthy sitting postures of a certain duration will be detected as unhealthy sitting postures.

### 4.3. Qualitative Analysis

As stated in Section 4.2, it is impossible to conduct comparison experiments with the auxiliary-equipment-based methods, the existing wearing-equipment-based methods and the vision-based methods on the same dataset. Therefore, only qualitative analysis is performed for these methods. The qualitative comparison is done in two aspects in terms of scene understanding and comprehensive features extraction, which substantially affect the correct detection of various types of unhealthy sitting postures.

On the one hand, the existing methods overlooked the understanding of scene relevance. The auxiliary-equipment-based methods [14,17,18] and the wearing-equipment-based methods [12,13] determined the unhealthy sitting postures with sensor devices such as pressure sensors and speed sensors. The vision-based methods [19,20] judged unhealthy sitting postures by visual information such as face information and skin color. The Kinect-based methods [23,24] judged unhealthy sitting postures by the key points of human body such as the torso angle and neck angle. All of the above features are extracted only from human body, lacking any understanding of relevancies among objects in the scene. In complicated scenes with multiple persons, multiple screens, multiple chairs and so on, the existing methods cannot exclude the extra persons and the extra screens because those methods don’t analyze and understand the relevancies among scenes. Therefore, these existing methods fail to detect unhealthy sitting postures in the above complicated environments, as shown in the experimental results in Figure 12. On the contrary, the unhealthy sitting postures in the above complicated scenes were correctly detected by our proposed method, as shown in Figure 12.

On the other hand, lacking comprehensive features extraction in the existing methods leads to missing detection of some types of unhealthy sitting postures. For the vision-based methods [19,20] using face motion and skin color statistics to detect sitting postures, occlusion may result in lost face information, and light intensity may affect the statistics of skin color. When a person bends over on the desk with complete occlusion of face, as shown in Figure 10e, these methods fail to detect faces and henceforth fail to detect this type of unhealthy sitting posture. The Kinect-based methods [23,24] using the key points of human body to detect sitting posture could not recognize screens and henceforth could not obtain the sight angle and sight distance. Therefore, these methods could neither detect the unhealthy sitting posture of excessive sight angle in looking up as shown in Figure 10f, nor detect the unhealthy sitting posture of too small sight distance in leaning forward as shown in Figure 10h.

## 5. Conclusions

In this paper, a scene recognition and semantic analysis approach to unhealthy sitting posture detection in screen-reading is proposed. The key skeletal points of human body are detected and tracked with a Microsoft Kinect sensor. Meanwhile, a deep learning method is used in the scene recognition of our method to accurately detect objects and extract relevant features. Then our method performs semantic analysis through Gaussian-Mixture behavioral clustering for scene understanding. The relevant features in the scene and skeletal features in human are fused into semantic features to discriminate various types of sitting postures. Our proposed method the problems of existing methods that only focus on extracting features of human themselves and lack understanding of the relevancies among objects in the scene. Experimental results demonstrated that our method accurately and effectively detected various types of unhealthy sitting postures in screen-reading and avoided error detection in complicated environments. Compared with the existing methods, our proposed method detected more types of unhealthy sitting postures that the existing methods could not detect. Detecting unhealthy sitting posture has far-reaching implications for medical assistance in health care or treatment in the workplace, and can also be applied to intelligent robotic systems to improve the life quality of humans. Behavior analysis through posture recognition is an important research topic in robotic systems. Our research is still in progress. We will conduct further exploration in the next step. Our proposed method can be potentially implemented on embedded pervasive platforms with the aim of enabling online/real-time posture detection so as to provide live feedback to users. We will further our research on this aspect in the future. We will also conduct further research on using more semantic features in our method in more complicated environments.

## Figures and Tables

**Table 1:** Comparison of multi-object detection methods based on deep learning.

Method	Device	Model	Region Proposal	Test (Region Proposal + Detection)
R-CNN	CPU & GPU	Mulit-ConvNet Multi-Classifier	Selective Search	2 s + 47 s
SPP-net	CPU & GPU	Mulit-ConvNet SPP Pooling	Selective Search	2 s + 2.3 s
Fast R-CNN	CPU & GPU	Share ConvNet RoI Pooling	Selective Search	2 s + 0.32 s
Faster R-CNN	GPU	End-to-End Share ConvNet RoI Pooling	Region Proposal Network	0.01 s+ 0.2 s

**Table 2:** The result of multi-object detection in Faster R-CNN.

Object	Real Result	Detection Result		Recall	Precision	Accuracy
		Positive	Negative			
Person	TRUE	2562	817	93.33%	85.40%	84.48%
	FALSE	438	183			
Chair	TRUE	2151	693	87.51%	71.70%	71.60%
	FALSE	849	307			
Screen	TRUE	2212	759	90.18%	73.73%	74.28%
	FALSE	788	241			

**Table 3:** The results of detecting unhealthy sitting posture in different methods based on self-collected dataset.

Different Sitting Postures	The Total Number of Videos	The Total Number of Detected as Unhealthy Sitting Posture	
		Kinect-Based Method Using Neck Angle and Torso Angle [24]	Our Proposed Method
Healthy sitting posture	65	6	2
Lean forward	65	59	62
Hold head	60	57	57
Lean backward	60	52	56
Bend over	60	58	58
Looking up	60	49	57
Body side	65	59	63
Too small sight distance	65	54	62

**Table 4:** Overall comparison of our method with some other methods.

Method	Features	Classifier	Dataset	*TP*	*TN*	*FP*	*FN*
Kinect-based method [24]	Torso angle, Neck angle	Threshold	Self-collected dataset (435 positive samples and 65 negative samples)	388	59	6	47
Our proposed method	Lumbar angle, Cervical angle, Sight angle, Sight distance, Spatial distance, Overlapping area	Scene Recognition using Faster R-CNN and Semantic Analysis using Gaussian-Mixture Model		**415**	**63**	**2**	**20**
